# Brain microdialysate tau dynamics predict functional and neurocognitive recovery after poor-grade subarachnoid haemorrhage

**DOI:** 10.1093/braincomms/fcac342

**Published:** 2023-01-02

**Authors:** Marina Heilig, Verena Rass, Anna Lindner, Mario Kofler, Bogdan-Andrei Ianosi, Maxime Gaasch, Lauma Putnina, Christian Humpel, Christoph Scherfler, Laura Zamarian, Thomas Bodner, Atbin Djamshidian, Alois Schiefecker, Claudius Thomé, Ronny Beer, Bettina Pfausler, Raimund Helbok

**Affiliations:** Department of Neurology, Medical University of Innsbruck, Innsbruck 6020, Austria; Department of Neurology, Medical University of Innsbruck, Innsbruck 6020, Austria; Department of Neurology, Medical University of Innsbruck, Innsbruck 6020, Austria; Department of Neurology, Medical University of Innsbruck, Innsbruck 6020, Austria; Department of Neurology, Medical University of Innsbruck, Innsbruck 6020, Austria; Department of Neurology, Medical University of Innsbruck, Innsbruck 6020, Austria; Department of Neurology, Medical University of Innsbruck, Innsbruck 6020, Austria; Department of Psychiatry and Psychotherapy, Laboratory of Psychiatry and Experimental Alzheimer’s Research, Medical University of Innsbruck, Innsbruck 6020, Austria; Department of Neurology, Medical University of Innsbruck, Innsbruck 6020, Austria; Department of Neurology, Medical University of Innsbruck, Innsbruck 6020, Austria; Department of Neurology, Medical University of Innsbruck, Innsbruck 6020, Austria; Department of Neurology, Medical University of Innsbruck, Innsbruck 6020, Austria; Department of Neurology, Medical University of Innsbruck, Innsbruck 6020, Austria; Department of Neurosurgery, Medical University of Innsbruck, Innsbruck 6020, Austria; Department of Neurology, Medical University of Innsbruck, Innsbruck 6020, Austria; Department of Neurology, Medical University of Innsbruck, Innsbruck 6020, Austria; Department of Neurology, Medical University of Innsbruck, Innsbruck 6020, Austria; Department of Neurology, Kepler University Hospital, Johannes Kepler University Linz, Linz, Austria

**Keywords:** subarachnoid haemorrhage, brain total tau, cerebral microdialysis, functional outcome, neurocognitive outcome

## Abstract

Subarachnoid haemorrhage is a devastating disease that results in neurocognitive deficits and a poor functional outcome in a considerable proportion of patients. In this study, we investigated the prognostic value of microtubule-associated tau protein measured in the cerebral microdialysate for long-term functional and neuropsychological outcomes in poor-grade subarachnoid haemorrhage patients. We recruited 55 consecutive non-traumatic subarachnoid haemorrhage patients who underwent multimodal neuromonitoring, including cerebral microdialysis. Mitochondrial dysfunction was defined as lactate-to-pyruvate ratio >30 together with pyruvate >70 mmol/L and metabolic distress as lactate-to-pyruvate ratio >40. The multidimensional 12-month outcome was assessed by means of the modified Rankin scale (poor outcome: modified Rankin scale ≥4) and a standardized neuropsychological test battery. We used multivariable generalized estimating equation models to assess associations between total microdialysate-tau levels of the first 10 days after admission and hospital complications and outcomes. Patients were 56 ± 12 years old and presented with a median Hunt & Hess score of 5 (interquartile range: 3–5). Overall mean total microdialysate-tau concentrations were highest within the first 24 h (5585 ± 6291 pg/mL), decreased to a minimum of 2347 ± 4175 pg/mL on Day 4 (*P* < 0.001) and remained stable thereafter (*P* = 0.613). Higher total microdialysate-tau levels were associated with the occurrence of delayed cerebral ischaemia (*P* = 0.001), episodes of metabolic distress (*P* = 0.002) and mitochondrial dysfunction (*P* = 0.034). Patients with higher tau levels had higher odds for a poor 12-month functional outcome (adjusted odds ratio: 2.61; 95% confidence interval: 1.32–5.17; *P* = 0.006) and impaired results in the trail making test-B (adjusted odds ratio: 3.35; 95% confidence interval: 1.16–9.68; *P* = 0.026) indicative of cognitive flexibility. Total microdialysate-tau levels significantly decreased over the first 10 days (*P* < 0.05) in patients without delayed cerebral ischaemia or good functional outcomes and remained high in those with delayed cerebral ischaemia and poor 12-month outcomes, respectively. Dynamic changes of total tau in the cerebral microdialysate may be a useful biomarker for axonal damage associated with functional and neurocognitive recovery in poor-grade subarachnoid haemorrhage patients. In contrast, ongoing axonal damage beyond Day 3 after bleeding indicates a higher risk for delayed cerebral ischaemia as well as a poor functional outcome.

## Teaser

Heilig *et al*. used cerebral microdialysis to quantify brain extracellular tau protein as a prognostic biomarker in poor-grade subarachnoid haemorrhage patients. As a marker for axonal injury, higher tau protein levels were associated with an unfavourable long-term functional and neuropsychological outcome after a non-traumatic subarachnoid haemorrhage.

## Graphical Abstract

**Graphical abstract:**
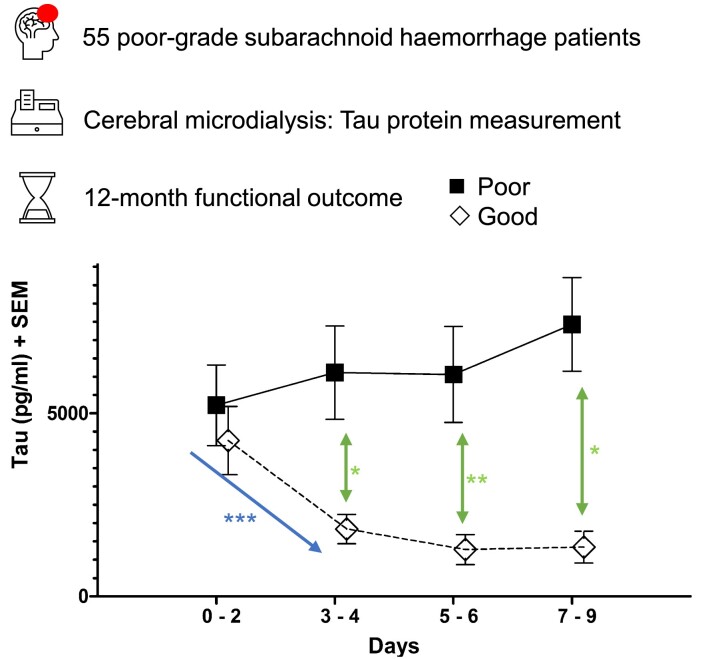

## Introduction

Non-traumatic subarachnoid haemorrhage (SAH) accounts for 5% of all strokes.^1^ Due to the relatively young age at disease onset^2^ and the high case fatality rates of up to 50%,^1^ the loss of potential life years is disproportionately high after SAH compared with other subtypes of stroke.^3^ Advances in neurocritical care have led to improved survival rates within the past decade, resulting in a higher proportion of patients with poor functional outcomes.^4^ Even patients with a relatively good functional outcome may suffer from long-term neurocognitive deficits.^5–7^ In particular, impairments in memory, executive functions and language are common in SAH survivors, frequently leading to loss of employment and diminished health-related quality of life.^6,8,9^

There is a need to early identify patients at risk of secondary brain injury as well as poor outcomes based on further characterization of underlying pathophysiologic mechanisms, where brain-derived biomarkers could be a useful tool. Both the extent of primary and secondary brain injuries are associated with worse outcomes after SAH. There is increasing evidence that early brain injury (EBI) within the first 72 h after bleeding is a valuable predictor for secondary brain injury, including delayed cerebral ischaemia (DCI).^10^ EBI and DCI share multifactorial underlying mechanisms such as autoregulatory dysfunction, cerebral oedema, microcirculatory dysfunction, excitotoxicity, neuroinflammation and cortical spreading depolarizations.^10,11^ Some of these mechanisms, together with increases in intracerebral pressure (ICP), lead to the disruption of the axonal integrity of the white matter.^12^ As a consequence, tau protein, a highly sensitive marker of axonal damage, is released into the brain’s extracellular compartment after SAH.^13^ Tau protein is predominantly located in neuronal axons, promotes microtubule assembly and stabilization and is further involved in axonal transport and signal transduction pathways.^14–19^ Cerebral microdialysis (CMD) as part of advanced multimodal neuromonitoring allows the measurement of brain extracellular metabolites.^20^ Accordingly, the microtubule-associated protein tau has been quantified in the brain extracellular fluid in SAH patients and was higher in patients with impaired brain metabolism, an unfavourable functional outcome and those with impaired neuropsychological test results.^13^ Similarly, higher CMD-tau levels have been associated with poor outcomes in patients with traumatic brain injury (TBI).^21^

In the current study, we intended to expand our previous observations^13^ in a larger patient population of consecutively recruited SAH patients and to specifically characterize the temporal profile of total CMD-tau dynamics. We hypothesized that brain extracellular levels of total tau protein decrease over time and that persistently higher levels are associated with both early and secondary brain injury as well as with poor long-term functional and neuropsychological outcomes after non-traumatic SAH.

## Materials and methods

### Study design, setting and participants

The study was designed according to the STrengthening the Reporting of OBservational studies in Epidemiology statement on observational cohort studies. We prospectively collected observational data on non-traumatic SAH patients admitted to the neurointensive care unit (NICU) of the Medical University of Innsbruck between April 2010 and September 2016 and performed a retrospective data analysis. Inclusion criteria were (i) age ≥18 years and (ii) invasive multimodal neuromonitoring, including CMD, as part of routine clinical care. Patients with a previous cerebrovascular disease or neurocognitive disease (as for example, dementia, ischaemic stroke, TBI or intracerebral haemorrhage), SAH due to arterio-venous malformation or length of intensive care unit stay <24 h were excluded from the study. Written informed consent, or deferred consent after regaining the capacity for consent, respectively, was obtained from all patients according to local regulations. The study protocol was approved by the local ethics committee (Medical University of Innsbruck, AN4091-292/4.6, AN3898-285/4.8) and was in accordance with the Declaration of Helsinki.

### Patient management and neurological grading

We graded patients clinically based on the Hunt & Hess (H&H) grade. Patient management followed current international guidelines,^22,23^ except for nimodipine, which was administered intravenously in poor-grade SAH patients. Ruptured aneurysms were treated either by endovascular coiling or neurosurgical clipping after multidisciplinary discussion. We performed transcranial colour-coded duplex sonography (TCCS) regularly to monitor for the development of large-vessel vasospasm. Vasospasm was defined as the elevation of mean TCCS-velocities >120 cm/s in the middle or anterior cerebral artery or daily changes of >50 cm/s in mean TCCS-velocities. In patients with severe vasospasm (>200 cm/s) refractory to induced hypertension, a catheter cerebral angiogram and treatment with intraarterial nimodipine were applied. DCI was diagnosed in the setting of neurological deterioration (a decrease of ≥2 points on the Glasgow coma scale), the occurrence of a new focal neurological deficit or a new cerebral infarction on CCT/MRI not attributable to other causes.^24^ Hospital complications, including DCI, were discussed in weekly meetings by the study team and treating intensivists. All patients were mechanically ventilated and received continuous administration of sufentanil, midazolam and/or ketamine.

### Neuromonitoring and data collection

Multimodal neuromonitoring was inserted in patients with (i) poor-grade SAH (HH scores: 4–5) or those with early (within 24 h of admission) neurological deterioration; (ii) expected prolonged mechanical ventilation and (iii) clinical or radiological signs suggestive of raised ICP as part of routine clinical care. Neuromonitoring probes included a parenchymal ICP probe (Neurovent-P-temp; Raumedic®, Helmbrechts, Germany), a brain tissue oxygen tension (P_bt_O_2_) probe (Licox® CC1.SB probes; Integra LifeSciences, Ratingen, Germany) and a high-cut-off (100 kDa) brain microdialysis catheter. They were inserted after aneurysm securement and placed into the hemisphere at the greatest risk of secondary brain injury (most commonly the frontal watershed ipsilateral to the aneurysm) either through a frontal burr hole using a triple-lumen bolt or tunnelled and placed in the white matter. The probe location was confirmed by cerebral CT immediately after insertion. For CMD monitoring, isotonic perfusion fluid (perfusion fluid CNS; M-Dialysis, Stockholm, Sweden) was pumped through the system at a flow rate of 0.3 μL/min. Samples from the first hour after probe insertion were discarded to avoid artefacts secondary to insertion trauma. Brain metabolic parameters, including glucose, lactate, pyruvate, and glutamate, were analysed immediately at the bedside. Afterwards, microdialysis samples were kept at −80°C. Mitochondrial dysfunction was defined as CMD-lactate-to-pyruvate ratio (LPR) >30 together with CMD-pyruvate >70 mmol/L, metabolic distress as LPR > 40, neuroglucopenia as CMD-glucose <0.7 mmol/L and brain metabolic crisis as CMD-glucose <0.7 mmol/L and CMD-LPR >40.

### Analytical methods

One microdialysis sample per day collected over 1 h was selected for every patient for the first 10 days after hospital admission. Analysis of total CMD-tau was performed by enzyme-linked immunosorbent assays as described previously.^13^ Briefly, 2 µL of undiluted microdialysis samples were added in singulates to the precoated plates and incubated overnight together with 100 µL of dilution buffer at 4°C, whereupon the wells were washed and the secondary antibody was added. After 30 min of incubation and another washing process, the substrate was added and the samples were incubated again for 10–30 min. Afterwards, absorbance was read on a microplate reader (Zenyth 3100) at 450 nm. All unknown samples were correlated to standards in duplicate. The detection limits were ∼40 pg/mL.

### Functional and neuropsychological outcome

We evaluated functional outcomes with the use of the modified Rankin scale (mRS) score three (telephone interview) and 12 months (in-person assessment by a physician of the study team whenever possible) after bleeding. We defined a good functional outcome as a mRS of 0–3 and a poor functional outcome as an mRS of 4–6. Five patients were lost to the 12-month follow-up and excluded from their respective analysis.

Additionally, detailed neuropsychological testing was performed in 20 of 45 survivors by trained neuropsychologists blinded to the clinical course of the patients 12 months after bleeding. The standardized neuropsychological test battery, included the screening of global cognitive functions (Mini-Mental State Examination; MMSE)^25^ and the assessment of verbal learning and memory (‘Verbaler Learn- und Merkfähigkeitstest’; VLMT), visual conceptualization and planning (executive clock drawing test , CLOX),^26^ psychomotor speed [trail making test (TMT)-A)],^27^ verbal attention span and verbal working memory [Wechsler memory scale (WMS) revised, digit span forwards]^28^ and cognitive flexibility and set-shifting ( TMT-B).^27^ As a global screening instrument for executive functions, the frontal assessment battery (FAB)^29,30^ was also applied.

The test results were dichotomized into normal and abnormal according to age and education scaled norms (VLMT, TMT-A, TMT-B, WMS: abnormal <10th percentile; FAB: abnormal <16th percentile), or predefined cut-off values (CLOX <10, MMSE <24).

### Statistical analysis

We defined the study time as the first 10 days after admission based on CMD availability to avoid selection bias. Day 0 conforms to the first 24 h after admission. Additionally, we performed statistical analysis using predefined periods: Days 0–2 including 107 samples, 2 [interquartile range (IQR), 2–3] per patient, Days 3–4 including 95 samples, 2 (IQR, 2–2) per patient, Days 5–6 including 88 samples, 2 (IQR, 2–2) per patient, Days 7–9 including 85 samples, 3 (IQR, 1–3) per patient.

We reported categorical variables as counts and proportions in each group. Continuous variables were assessed for normality using the Shapiro–Wilk test. Normally distributed data were given as mean and standard deviation (SD) or standard error of the mean (SEM) for graphics, whereas non-parametric data were given as median and IQR. Brain metabolic parameters (CMD-glucose, CMD-lactate and CMD-pyruvate) as well as other neuromonitoring parameters [ICP; cerebral perfusion pressure (CPP); P_bt_O_2_] were averaged over 24 h and matched to a CMD-tau sample period of 1 h. Spearman’s correlation was used to assess correlations between these monitoring parameters and CMD-tau levels. Time-series data were analysed using generalized estimating equations (GEEs), with the correlation matrix best fitting the model. CMD-tau data were logarithmized to meet assumptions for normality. To assess the association of CMD-tau with functional and neuropsychological outcome variables, the latter were dichotomized and used as a dependent variable in the binary logistic GEE model. The same method was applied to analyse the association between CMD-tau levels and episodes of brain metabolic changes. For multivariable binary logistic GEE models with DCI or functional outcome as dependent variable, variables with a *P* < 0.1 in univariate analysis (Fisher’s exact test, *χ*^2^ test or Mann–Whitney *U*-test as appropriate; Supplementary Tables 1 and 2) were included and kept if significant. Variables that were not significant were removed stepwise, except for the H&H score on admission, age and sex, which were kept in the models.

All other GEE models were adjusted for disease severity on admission (assessed with the H&H score), age and sex. Optimal cut-off levels of CMD-tau to predict 12-month functional outcome were calculated using receiver-operating characteristics (ROCs) minimizing the ‘distance closest to the top left corner’ of the ROC curves.^31^

For all tests, the significance level was set at a *P*-value <0.05. All analyses were performed with IBM-SPSS V27.0 for Windows (IBM Corp., Armonk, NY, USA).

## Results

### Patient characteristics

Out of 85 non-traumatic SAH patients undergoing multimodal neuromonitoring, 55 patients with CMD were included in the final analysis. Detailed information on demographics and clinical characteristics, hospital complications and outcomes is provided in Table 1.

**Table 1:** Demographical and clinical characteristics, neuromonitoring and outcome characteristics

Demographics and medical history (*N* = 55)		
Age (years)	56	±12
Sex (female)	39	71
History of smoking	27	49
Premedical history of hypertension	18	33
Diabetes mellitus II	6	11
Loss of consciousness at ictus	36	65
**Clinical findings on admission**		
Hunt& Hess grade		
1 and 2	2	4
3	16	29
4	7	13
5	30	54
Modified Fisher’s scale		
1	2	4
2	4	7
3	14	25
4	35	64
SEBES		
0	5	10
1	7	13
2	8	15
3	16	31
4	16	31
Aneurysm size >10 mm	13	25
**Aneurysm treatment**		
Clipping	39	71
Coiling	15	27
Non-aneurysmal SAH	1	2
**Hospital complications**		
Hyodrocephalus treated with EVD	41	74
Large-vessel cerebral vasospasm	44	80
DCI	16	29
Ventriculitis	7	13
Pneumonia	39	71
**Outcome characteristics**		
NICU LOS (days)	30	(23–46)
In-hospital mortality	6	11
Modified Rankin scale (after 3 months)		
0	1	2
1	7	13
2	10	18
3	7	13
4	8	14
5	14	25
6	8	14
Modified Rankin scale (after 12 months)		(*n* = 50)
0	7	14
1	10	20
2	11	22
3	1	2
4	6	12
5	5	10
6	10	20

Values are presented as mean ( ±SD), median (IQR) or count (%).

CMD, cerebral microdialysis; EVD, external ventricular drain; LOS, length of stay; NICU, neurointensive care unit; SAH, subarachnoid haemorrhage; SEBES, subrachnoid haemorrhage early brain oedema score.

On admission, two-thirds (*n* = 37) presented with a poor clinical grade (H&H scores: 4–5), while the remaining patients (*n* = 18) deteriorated within 24 h and required multimodal neuromonitoring as the standard of care. Invasive neuromonitoring was started at a median of 8 (IQR: 2–16) h after admission. The first CMD-tau sample used for analysis was obtained at a median of 10 (IQR, 5–23) h after admission. Overall, 375 tau samples with a median of 7 (IQR, 5–9) CMD-tau samples per patient were analysed within the first 10 days after admission. Hospital mortality was 11% (*n* = 6) and another 7% of patients (*n* = 4) died after hospital discharge within the first year after SAH.

### Temporal profile of mean total CMD-tau levels and associations with brain metabolic parameters

**Figure 1:**
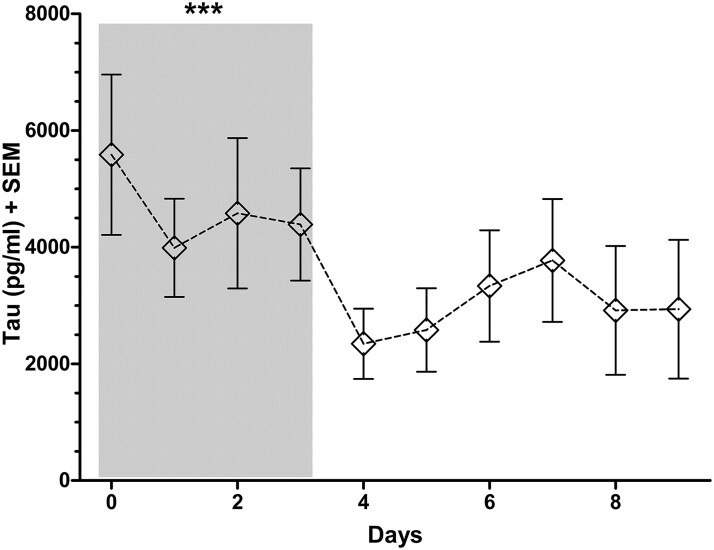
**Overall temporal profile of CMD-tau levels in SAH patients.** CMD-tau levels are presented as mean (SEM) values. Statistical analysis was performed using a linear GEE model. ***P = 0.001. The number of data points for each time period analysed are as follows: Days 0–2: *n* = 107; Days 3–4: *n* = 95, Days 5–6: *n* = 88, Days 7–9: *n* = 85.

The mean total CMD-tau concentrations were highest within the first 24 h (5585 ± 6291 pg/mL), decreased to a minimum of 2347 ± 4175 pg/mL on Day 4 (*P* < 0.001) and remained stable thereafter (*P* = 0.613; Fig. 1). CMD-tau was not associated with patients’ age (*P* = 0.368).

There was a significant correlation between CMD-tau and CMD-LPR (*r* = 0.43, *P* < 0.001), CMD-lactate (*r* = 0.46, *P* < 0.001), CMD-glutamate (*r* = 0.40, *P* < 0.001) and a weaker correlation with CMD-glucose (*r* = 0.17, *P* = 0.003) and CMD-pyruvate (*r* = 0.16, *P* = 0.006). No correlations were found between CMD-tau concentrations and other neuromonitoring parameters, including mean ICP (*P* = 0.422), mean CPP (*P* = 0.429) or mean P_bt_O_2_ (*P* = 0.875).

**Figure 2:**
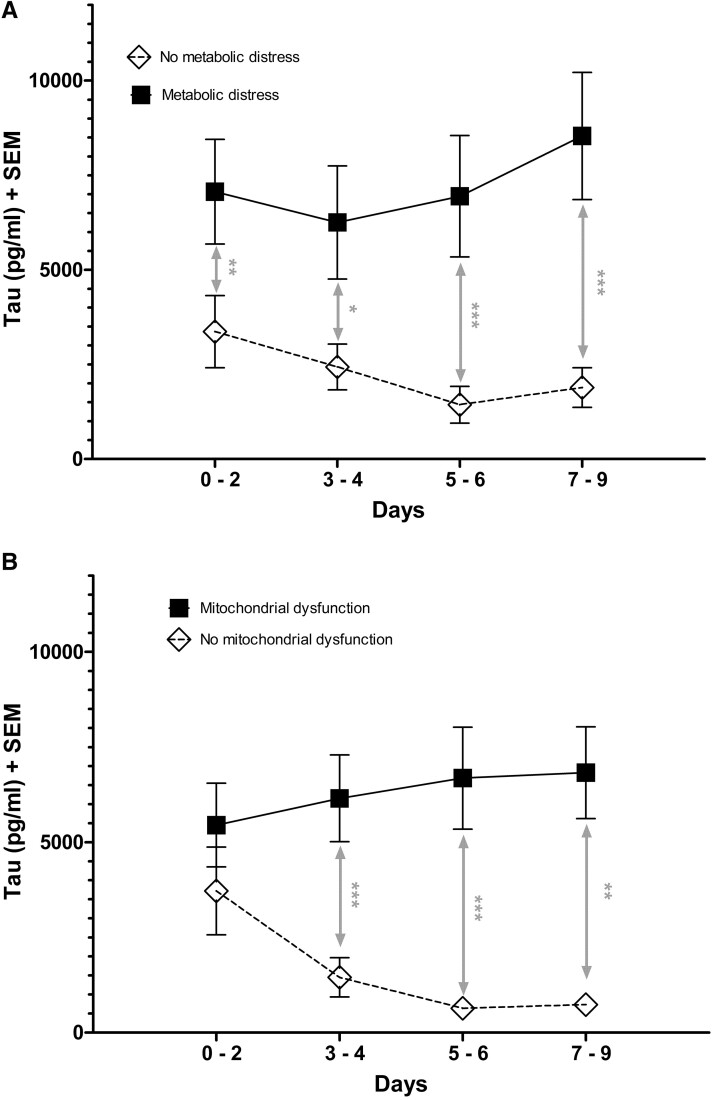
**CMD-tau levels and associations with brain metabolic parameters.** Temporal profile of mean (SEM) CMD-tau levels in patients with (▪) and without (□) (**A**) episodes of metabolic distress (***Days 0–2: *P* = 0.009, *n* = 56/25; Days 3–4: *P* = 0.012, *n* = 60/21; Days 5–6: *P* < 0.001, *n* = 51/24; Days 7–9: *P* < 0.001, *n* = 55/18), or (**B**) episodes of mitochondrial dysfunction (Days 0–2: *n* = 44/37; ***Days 3–4: *P* = 0.001, *n* = 47/34; Days 5–6: *P* < 0.001, *n* = 44/32; Days 7–9: *P* = 0.002, *n* = 41/32). Statistical analysis was performed using a binary logistic linear GEE adjusted for age, sex and H & H score on admission. *p < 0.05; **p < 0.01; ***p < 0.001.

Total CMD-tau levels were higher during episodes of brain metabolic distress [adjusted odds ratio (adj. OR): 1.97; 95% confidence interval (CI): 1.32–2.95; *P* = 0.001] and mitochondrial dysfunction (adj. OR: 1.39; 95% CI: 1.03–1.88; *P* = 0.033; Fig. 2). No associations were found between CMD-tau and neuroglucopenia (*P* = 0.413) or brain metabolic crisis (*P* = 0.111). While CMD-tau levels decreased significantly in patients without episodes of brain metabolic distress (adj. OR: 0.91; 95% CI: 0.87–0.96; *P* = 0.001) or mitochondrial dysfunction (adj. OR: 0.94, 95% CI: 0.89–0.98; *P* = 0.007), there was no significant change in CMD-tau levels over the study period in patients with episodes of brain metabolic distress (*P* = 0.783) or mitochondrial dysfunction (*P* = 0.843; Fig. 2).

### CMD-tau levels and early and secondary brain injury

We did not find a significant association between CMD-tau levels and markers of EBI, including the H & H score (*P* = 0.074), Fisher (*P* = 0.056) or high-grade subarachnoid haemorrhage early brain oedema score (SEBES 3–4, *P* = 0.333) on admission. There was also no significant difference in CMD-tau levels in patients who underwent neurosurgical clipping compared with those who underwent neuroradiological coiling (*P* = 0.435).

**Figure 3:**
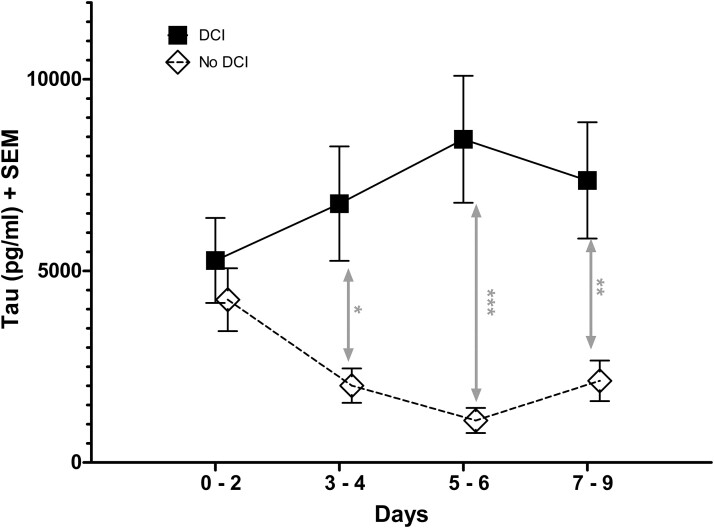
**CMD-tau levels and DCI.** Temporal profile of mean (SEM) CMD-tau levels in patients presenting with (▪) and without (□) DCI during the hospital course (Days 0–2, *n* = 77/30; ***Days 3–4: *P* = 0.012, *n* = 68/27; Days 5–6: *P* < 0.001, *n* = 66/22; Days 7–9: *P* = 0.003, *n* = 66/19). Statistical analysis was performed using a binary logistic linear GEE model adjusted for age, sex and H & H score on admission. *p < 0.05; **p < 0.01; ***p < 0.001.

However, higher CMD-tau levels were associated with the occurrence of DCI (adj. OR: 2.87, 95% CI: 1.53–5.38; *P* = 0.001). Regarding predefined time periods, this association did only apply for CMD-tau levels on Days 3–9 (adj. OR: 3.98, 95% CI: 2.00–7.91; *P* < 0.001), but not for CMD-tau levels on Days 0–2 (*P* = 0.389). Interestingly, CMD-tau levels remained constantly elevated in patients who experienced DCI (*P* = 0.365) but decreased significantly in patients without DCI (adj. OR: 0.93; 95% CI: 0.89–0.97; *P* = 0.001; Fig. 3).

### CMD-tau levels, mortality and functional outcome

**Figure 4:**
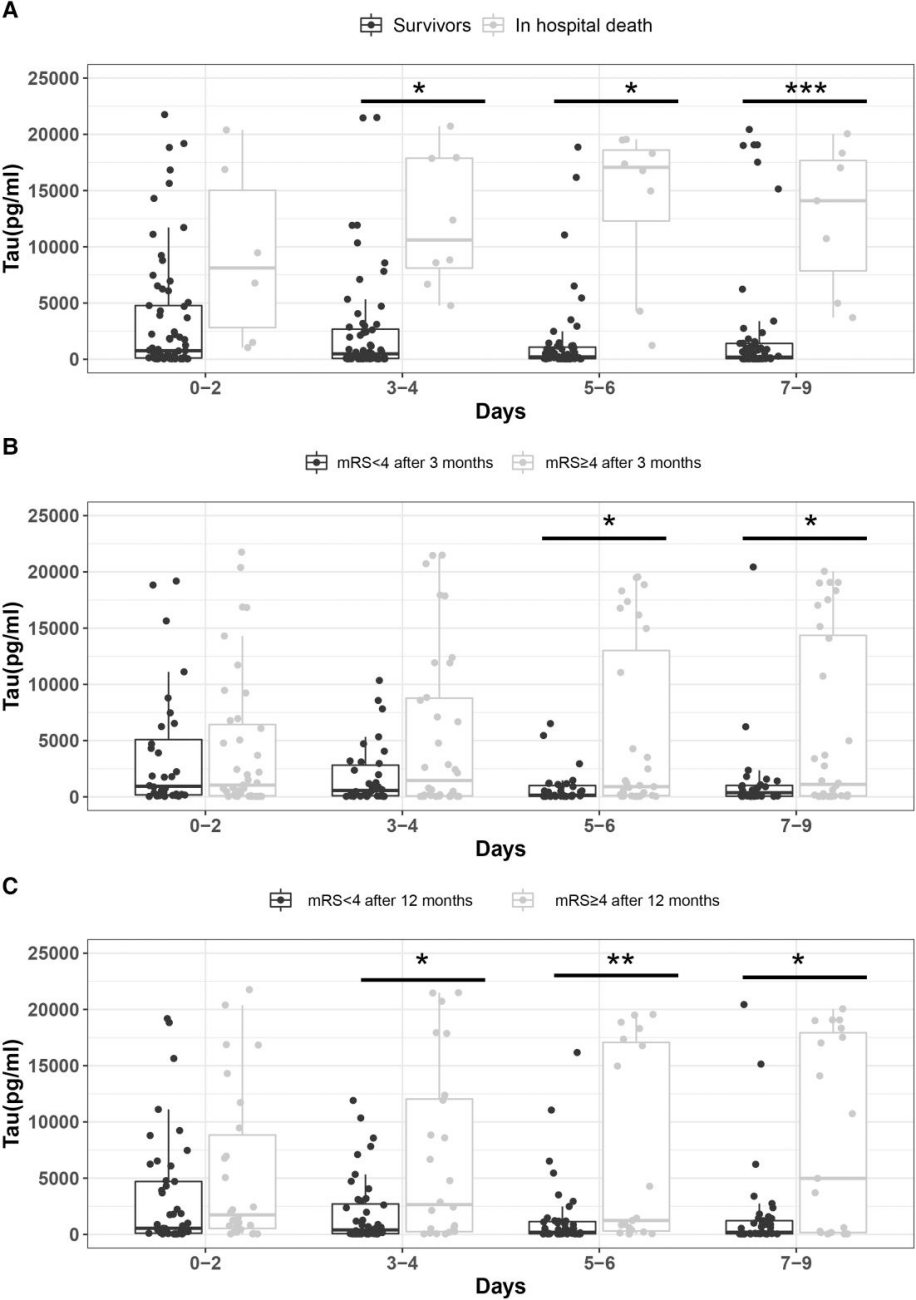
**CMD-tau levels and mortality and functional outcomes.** Temporal profile of median (IQR) CMD-tau levels in patients (**A**) who died during hospital course (▪) and those who survived during hospital course (□) (***Days 3–4: *P* = 0.014; Days 5–6: *P* = 0.045; Days 7–9: *P* = 0.001), (**B**) with mRS 4–6 (▪) and mRS 0–3 (□) at 3 months follow-up (***Days 5–6: *P* = 0.018; Days 7–9: *P* = 0.041) and (**C**) 12 months follow-up (***Days 3–4: *P* = 0.011; Days 5–6: *P* = 0.004; Days 7–9: *P* = 0.012). The central line shows the 50th percentile, the upper and lower lines show the 75th and 25th percentiles, and the vertical lines show the 90th and 10th percentiles. Statistical analysis was performed using a binary logistic linear GEE model adjusted for age, sex and H&H score on admission. *p < 0.05; **p < 0.01; ***p < 0.001.

In patients who died during hospitalization, total CMD-tau levels were higher (adj. OR: 6.21, 95% CI: 1.60–24.09; *P* = 0.008) and even increased over the neuromonitoring period (adj. OR: 1.09; 95% CI: 1.04–1.15; *P* = 0.001) compared with hospital survivors. In survivors, CMD-tau levels decreased (adj. OR: 0.94; 95% CI: 0.91–0.98; *P* = 0.003; Fig. 4A).

Around 3 months after SAH, 25 (45%) patients had good functional recovery and 30 (55%) patients experienced a poor functional outcome. At the 12-month follow-up, 29 patients (58%) showed good functional recovery, while 21 patients (42%) were assessed with a bad functional outcome.

CMD-tau levels between Days 3 and 9 were higher in patients with a poor 3-month functional outcome (adj. OR: 2.24, 95% CI: 1.12–4.48; *P* = 0.023) compared with those with a favourable outcome. However, this relationship was not true for the entire study period (*P* = 0.100). CMD-tau levels dropped significantly in patients who had a good 3-month functional outcome (adj. OR: 0.93, 95% CI: 0.87–1.00; *P* = 0.047) and remained elevated in patients with a poor 3-month functional outcome (*P* = 0.706; Fig. 4B).

Similarly, CMD-tau levels were significantly higher during the study time in patients with poor 12-month functional outcomes (adj. OR: 2.58; 95% CI: 1.34–4.96; *P* = 0.004) compared with patients with good outcomes. Again, we found a significant decrease of CMD-tau over the first 10 days in patients with a good 12-month functional outcome (adj. OR: 0.91; 95% CI: 0.87–0.95; *P* < 0.001), with no change in patients with a poor 12-month functional outcome (*P* = 0.391; Fig. 4C).

The mean CMD-tau values derived from Day 6 showed the best ability to discriminate between good and poor functional outcomes at 12 months with an area under the receiver operating characteristic of 0.800. The best cut-off was at a CMD-tau value of 515 pg/mL with 79% sensitivity and 74% specificity.

### CMD-tau levels and neuropsychological outcome

Of the 45 survivors, 20 finalized neuropsychological testing at the 1-year follow-up. Of these, 14 patients (70%) showed neuropsychological deficits in at least one neuropsychological test, most frequently in the VLMT (55%), FAB (40%) and TMT-A (40%, Table 2). Importantly, all of these 20 patients showed good functional recovery (mRS < 4) at the 12-month follow-up. Higher CMD-tau levels during the study time were associated with TMT-B < 10th percentile (adj. OR: 3.35; 95% CI: 1.16–9.68; *P* = 0.026). There was no association between CMD-tau levels and FAB (*P* = 0.582), TMT-A (*P* = 0.309) or VLMT (*P* = 0.127). Almost all patients achieved normal test results in the WMS, MMSE, CLOX and statistics therefore did not apply.

**Table 2:** Neuropsychological outcomes at the 12-month follow-up in 20 patients

Neuropsychological tests		*n*	%
CLOX	<10	0	0
FAB	<16th percentile	8/20	40
MMSE	<24	2/20	10
TMT-A	<10th percentile	8/20	40
TMT-B	<10th percentile	6/16	38
VLMT	<10th percentile	11/20	55
WMS	<10th percentile	3/19	16

Values are presented as count (percentage).

CLOX, clock drawing test; FAB, assessment battery; MMSE, Mini-Mental State Examination score; TMT, Trail making test; VLMT, Verbaler Learn- und Merkfähigkeitstest; WMS, Wechsler memory scale.

## Discussion

In line with our previous study,^13^ we found that elevated CMD-tau levels during the acute phase were significantly associated with episodes of metabolic distress, episodes of mitochondrial dysfunction and worse functional and neuropsychological outcomes 3–12 months after non-traumatic SAH. Furthermore, persistently elevated CMD-tau levels were associated with the occurrence of DCI as well as poor functional and neurocognitive outcomes. Interestingly, a decrease of initially elevated CMD-tau levels was indicative for improved cellular homeostasis as well as functional recovery over the long-term.

Biomarkers are useful to gain information on cellular, biochemical or molecular mechanisms involved in neuronal injury and repair mechanisms after acute brain injury and thereby may add diagnostic and prognostic information in addition to neuroimaging and clinical markers of disease severity. Increased levels of tau protein in the cerebrospinal fluid (CSF) or serum as a marker of axonal injury^32–34^ have been associated with poor outcomes and hospital complications in patients with spontaneous SAH,^34^ TBI^35,36^ and ischaemic stroke.^37,38^ Tau protein measured in the brain extracellular level derived from CMD may be even more specific since Tau is primarily localized in neuronal axons and released into the extracellular space due to acute brain injury and dilutional effects are avoided.^13,21^ Whether CMD-tau, derived from the local brain environment at greatest risk of secondary injury, provides more diagnostic and prognostic accuracy compared with CSF-tau levels, needs to be elucidated in a comparative study including CMD-tau and CSF-tau levels.

We found elevated CMD-tau levels, especially in the initial phase after SAH, with stabilization after Day 4. The observed temporal profile most likely represents axonal injury or disturbed axonal integrity, which may be most prevalent in the initial phase after SAH, secondary to mechanisms of EBI, including increased ICP and decreased CPP with concomitant neuronal cell loss.^10^ This hypothesis is further supported by the association between elevated CMD-tau levels and impaired brain metabolism, including mitochondrial dysfunction and metabolic distress. Apart from increased ICP, underlying pathophysiological mechanisms of axonal injury include ischaemic and non-ischaemic aetiologies secondary to energy impairments associated with mitochondrial dysfunction or cortical spreading depolarizations.^39^ In a previous study, we could show that increased CMD-tau levels were associated with higher IL-6 levels in SAH patients, suggesting that neuroinflammation contributes to axonal injury.^40^ Similarly, blood degradation products such as iron were trapped in the cerebral white matter after SAH, as assessed with CMD^41^ or advancing imaging.^42^ They remained elevated at the 1-year follow-up imaging and were associated with neuropsychological deficits.^42^ Understanding the pathophysiology is important because white matter injury is potentially reversible in contrast to grey matter injury, where neuronal apoptosis occurs.^12^

All these mechanisms are considered to be mitigators of EBI and DCI.^10,11^ Interestingly, we could not demonstrate an association between CMD-tau levels and clinical or radiographic markers of EBI, most likely owing to the fact that all of our patients were poor-grade patients or deteriorated within the first 24 h leading to the indication of invasive multimodal neuromonitoring. Importantly, we found that higher CMD-tau levels were associated with the occurrence of DCI. There is increasing evidence that the extent of EBI is associated with the development of DCI.^11^ On the other hand, prolonged release of tau may directly result from secondary brain injury, which is supported by the relatively short half-life (115 h) of tau protein.^43^ Literature on tau dynamics and sustained tau elevation in SAH patients is rare. A study investigating CSF-tau demonstrated a delayed increase in patients with clinically evident vasospasm, which confirms our finding.^34^

Although we discarded the first hour of CMD measurements to avoid artefacts of insertion trauma, we cannot exclude its relevance in the interpretation of the initial peak. Still, we found distinct patterns of the temporal profile of CMD-tau levels over the study period of 10 days, arguing against a significant effect of the initial insertion trauma. Furthermore, total tau levels decreased in patients without DCI or good functional outcome, but remained constantly high in those with secondary brain injury and poor functional outcome. This underlines the hypothesis of ongoing axonal injury in poor-grade SAH patients for several days after the initial bleeding, with continuous release of tau from injured neurons. In line with the prolonged release of tau possibly resulting from secondary brain injury, we found that the highest discriminative power of increased CMD-tau to predict outcome was on Day 6, where the difference of tau dynamics became more relevant. In a previous magnetic resonance imaging (MR) study conducted in good-grade SAH patients, we found new microstructural axonal damage at the 12-month follow-up when compared with the 3-week time point, which may suggest prolonged neuronal and axonal injury even beyond the acute phase.^42^ A recent study in TBI patients could demonstrate that the persistence of anti-MAG (Myelin-associated glycoprotein) IgM autoantibodies over the first year in some patients was correlated with raised serum neurofilament light levels, which is another marker of axonal damage.^44^ This would not only suggest an association between autoantibodies and ongoing white matter tract degeneration over the first year post-injury but also raise the question of whether axonal injury after SAH may contribute to long-term cognitive decline and dementia. From epidemiological studies, we know that SAH survivors are at higher risk for dementia compared with the general population.^13^ On a molecular basis, axonal injury may cause up-regulation of kinases promoting post-translational tau modifications and therefore may prime neurons for neurodegeneration.^45^ A prospective multi-centre cohort study, namely the DISCOVERY trial,^46^ is currently underway investigating long-term cognitive outcomes after ischaemic and haemorrhagic stroke including SAH patients. The study aims to elucidate mechanisms of brain resilience and susceptibility to post-stroke cognitive impairment and dementia using advanced neuroimaging and comprehensive genetic/genomic and fluid biomarker testing.

We found an association between elevated CMD-tau levels and poor functional outcomes comparable with previous reports in SAH^13,34^ and TBI patients.^21^ This finding again emphasizes the association between extracellular tau protein and axonal injury, since a higher degree of axonal injury with loss of central white matter axons determines neurological damage. Interestingly and in contrast to patients with good functional recovery, CMD-tau levels did not decrease in patients with an unfavourable functional outcome. This finding further supports the importance and the additional prognostic value of a longitudinal analysis of tau protein after SAH.

Moreover, elevated CMD-tau levels during the acute phase were indicative of an impaired neurocognitive outcome one year after the initial bleeding. This is important as all patients with neuropsychological deficits were classified as having a good functional outcome (mRS <4) emphasizing that even patients with a good outcome suffer from long-term cognitive changes after SAH. Therefore, cognitive testing should be integrated in the follow-up of SAH patients in order to provide individualized treatment plans such as neurocognitive training. Promising neuroprotective treatment approaches to target white matter injury are not yet ready for clinical practice.^12^ To date, clinicians should focus on vigilant monitoring of secondary brain injury to prevent or alleviate it by, i.e. means of targeted temperature management or adequate energy supply during the acute phase.^11^

Although we were confronted with a selection bias and only patients with a good functional 1-year outcome were systematically assessed for neuropsychological deficits, we found an association between higher tau levels and impaired test results in the TMT-B indicative of impairment in cognitive flexibility and set-shifting. In accordance with the current results, in a previous MR study, we could show that axonal damage adjacent to the left middle and superior frontal gyrus and the left supplementary area was associated with impairments in executive functions.^42^

Some limitations of the study deserve to be discussed. Firstly, the analysis was limited to tau levels within the brain extracellular fluid and we cannot compare our results to previous studies quantifying CSF-tau levels. However, we think that localized measurements directly in the white matter are more precise to provide insights into brain pathophysiology in contrast to CSF reflecting the central nervous system as a whole.^47^ Secondly, due to ethical reasons, we could not include good-grade patients or a healthy control group. Extracellular concentrations of the intracellular cytoskeletal tau protein^32,33^ are expected to be low under normal conditions, which has already been demonstrated in CSF studies.^34,48^ In this regard, we may have even underestimated our findings, as only poor-grade patients were compared. Still, the data underlie a selection bias and the results may not be transferable to good-grade patients. Thirdly, the observational study design does not allow us to prove a causal relationship between axonal injury and elevated CMD-tau levels. Despite this, our data strongly support the hypothesis of higher extracellular tau concentrations in patients with acute brain injury. Last, data interpretation is limited by the size of our patient cohort. Especially the link between elevated CMD-tau levels and impaired neuropsychological test results should regarded as hypothesis generating, since a considerable proportion of patients could not undergo detailed neuropsychological testing due to a poor clinical status at the 1-year follow-up.

## Conclusion

Our study demonstrates that dynamic changes of CMD-tau levels in poor-grade SAH patients may be useful as a biomarker for axonal damage secondary to primary and secondary brain injury mechanisms and may help to predict survival and long-term neurological outcomes. Decreases of tau may be representative of recovery, while sustained high levels might be indicative of persistent axonal damage from secondary injury associated with poor outcome.

## Supplementary Material

fcac342_Supplementary_Data

## Data Availability

The data that support the findings of this study are available from the corresponding author, upon reasonable request and fulfilling data sharing regulations approved by the local ethics committee.

## Abbreviations

CLOX =: clock drawing test
CMD =: cerebral microdialysis
CSF =: cerebrospinal fluid
DCI =: delayed cerebral ischaemia
EBI =: early brain injury
FAB =: frontal assessment battery
GCS =: Glasgow coma scale
GEE =: general estimating equation
H&H =: Hunt & Hess
ICP =: intracranial pressure
ICU =: intensive care unit
IQR =: interquartile range
LPR =: lactate pyruvate ratio
MMSE =: mini-mental state examination
mRS =: modified Rankin scale
ROC =: receiver-operating characteristics
SAH =: subarachnoid haemorrhage
SD =: standard deviation
SEBES =: subarachnoid haemorrhage early brain oedema score
SEM =: standard error of mean
TCCS =: transcranial colour-coded duplex sonography
TMT =: trail making test
WMS =: Wechsler memory scale
