# Supplementary material for: Brain microdialysate tau dynamics predict functional and neurocognitive recovery after poor-grade subarachnoid haemorrhage
Source: Brain Commun. 2023 Jan 2;5(1):fcac342. doi: 10.1093/braincomms/fcac342 (PMC9851418; doi:10.1093/braincomms/fcac342)
Supplement: fcac342_Supplementary_Data [file fcac342_supplementary_data.docx]

**Supplemental Table 1. Factors associated with functional outcome at the 12-months follow-up**

| **mRS at 12 Months** | **0-3**  **(n = 29)** | | **4-6**  **(n =21)** | | **Univariate P-Value** | **Multivariable OR (95%CI), P-Value** |
| --- | --- | --- | --- | --- | --- | --- |
| **Demographics and Past Medical History** | | | | | |  |
| Age (years)***** | 53 | [± 11] | 60 | [± 12] | **0.023** | 1.105 (1.026-1.189), P = 0.008 |
| Sex (female) | 21 | (72) | 13 | (62) | 0.543 | 0.338 (0.073-1.567), P = 0.166 |
| History of smoking | 15 | (52) | 11 | (52) | 1.000 |  |
| Premedical history of hypertension | 10 | (35) | 7 | (33) | 1.000 |  |
| Diabetes mellitus II | 1 | (3) | 2 | (10) | 0.565 |  |
| Loss of consciousness at ictus | 17 | (59) | 15 | (71) | 0.388 |  |
| **Clinical Findings on Admission** | | | | | |  |
| Hunt-Hess-Grade on admission***** | 4 | [3-5] | 5 | [4-5] | **0.043** | 2.201 (1.069-4.531), P = 0.032 |
| Modified Fisher Scale on admission | 4 | [3-4] | 4 | [3-4] | 0.250 |  |
| SEBES on admission | 3 | [2-4] | 3 | [1-3] | 0.177 |  |
| Aneurysm size >10 mm***** | 4 | (14) | 9 | (43) | **0.022** |  |
| Anterior vs. posterior circulation aneurysm | 24/5 | (83/17) | 19/1 | (95/5) | 0.379 |  |
| **Aneurysm treatment** | | | | | |  |
| Clipping/Coiling/No intervention***** | 18/11/0 | (62/38/0) | 18/2/1 | (85/10/5) | **0.047** |  |
| Hemicraniectomy | 1 | (3) | 1 | (5) | 1.000 |  |
| **Hospital Complications** | | | | |  |  |
| Hydrocephalus treated with EVD | 22 | (76) | 15 | (71) | 0.754 |  |
| Large-vessel cerebral vasospasm | 21 | (72) | 18 | (86) | 0.319 |  |
| Delayed cerebral ischaemia***** | 5 | (17) | 10 | (48) | **0.030** |  |
| Ventriculitis | 5 | (17) | 2 | (10) | 0.684 |  |
| Pneumonia | 21 | (72) | 15 | (71) | 1.000 |  |
| CMD-Tau |  |  |  |  |  | 2.581 (1.343-4.960), P = 0.004 |
| Values are presented as mean [± SD], median [IQR] or count (%).  *****P < 0.05 (significant difference between patients with good and poor functional outcome at 12-months follow-up, univariate analysis was performed with Exact Fisher Test or Chi² Test for categorical variables and Mann-Whitney-U-Test for continuous variables).  mRS = Modified Rankin Scale, SEBES = Subarachnoid Haemorrhage Early Brain Edema Score, EVD = External Ventricular Drain, CMD = Cerebral Microdialysis | | | | | |  |

**Supplemental Table 2. Factors associated with the occurrence of DCI during the hospital course**

| **Occurrence of DCI** | **No**  **(n = 39)** | | **Yes**  **(n =16)** | | **Univariate P-Value** | **Multivariable OR (95%CI), P-Value** |
| --- | --- | --- | --- | --- | --- | --- |
| **Demographics and Past Medical History** | | | | | |  |
| Age (years) | 56 | [±12] | 57 | [±12] | 0.874 | 1.021 (0.960-1.085), P = 0.514 |
| Sex (female) | 30 | (77) | 9 | (56) | 0.191 | 0.296 (0.076-1.159), P = 0.080 |
| History of smoking | 19 | (49) | 8 | (50) | 1.000 |  |
| Premedical history of hypertension | 12 | (31) | 6 | (38) | 0.754 |  |
| Diabetes mellitus II | 5 | (13) | 1 | (6) | 0.660 |  |
| Loss of consciousness at ictus | 25 | (64) | 11 | (69) | 1.000 |  |
| **Clinical Findings on Admission** | | | | | |  |
| Hunt-Hess-Grade on admission***** | 4 | [3-5] | 5 | [4-5] | **0.034** | 2.669 (1.211-5.884), P = 0.015 |
| Modified Fisher Scale on admission | 4 | [3-4] | 4 | [3-4] | 0.367 |  |
| SEBES on admission | 3 | [2-4] | 3 | [2-3] | 0.843 |  |
| Aneurysm size >10 mm | 8 | (21) | 5 | (31) | 0.493 |  |
| Anterior vs. posterior circulation | 34/4 | (90/10) | 14/2 | (88/12) | 1.000 |  |
| **Aneurysm treatment** | | | | | |  |
| Clipping/Coiling/No intervention | 28/10/1 | (26/71/3) | 11/5/0 | (69/31/0) | 0.747 |  |
| Hemicraniectomy | 1 | (3) | 1 | (6) | 0.501 |  |
| **Hospital Complications** | | | | |  |  |
| Hydrocephalus treated with EVD | 29 | (74) | 12 | (75) | 1.000 |  |
| Ventriculitis | 6 | (15) | 1 | (6) | 0.660 |  |
| Pneumonia***** | 32 | (82) | 7 | (44) | **0.008** |  |
| CMD-Tau |  |  |  |  |  | 2.871 (1.533-5.376), P = 0.001 |
| Values are presented as mean [± SD], median [IQR] or count (%).  *****P < 0.05 (significant difference between patients with and without DCI, univariate analysis was performed with Exact Fisher Test or Chi² Test for categorical variables and Mann-Whitney-U-Test for continuous variables).  DCI = Delayed Cerebral Ischaemia, SEBES = Subarachnoid Haemorrhage Early Brain Edema Score, EVD = External Ventricular Drain, CMD = Cerebral Microdialysis | | | | | |  |
